# Cochlear macrophage CD74 enhances the apoptosis of senescent cochlear hair cells by down-regulating MIF

**DOI:** 10.3389/fimmu.2026.1751126

**Published:** 2026-03-10

**Authors:** Xiao-Mei Sun, Qi-Yang Sun, Meng-Qi Zhao, Wei-Ping Wen, Fan-Qin Wei

**Affiliations:** 1 Department of Otorhinolaryngology-Head and Neck Surgery, The Sixth Affiliated Hospital, Sun Yat-sen University, Guangzhou, Guangdong, China; 2 Biomedical Innovation Center, The Sixth Affiliated Hospital, Sun Yat-sen University, Guangzhou, Guangdong, China; 3 Department of Otorhinolaryngology-Head and Neck Surgery, The First Affiliated Hospital, Sun Yat-sen University, Guangzhou, Guangdong, China

**Keywords:** age-related hearing loss, CD74, hair cells, macrophages, MIF

## Abstract

Age-related hearing loss is a global public health issue that impacts the quality of life in the elderly population. Macrophages are the main immune cell population in the cochlea, yet the role in the development and progression of age-related hearing loss is still unclear. This study analyzed single-cell sequencing data from cochlear tissues of C57BL/6J mice across different ages and identified notable increase in CD74 expression in macrophages with aging. Validation revealed that CD74 levels were elevated in the aged cochlea, while macrophage migration inhibitory factor (MIF) levels decreased. MIF was significantly reduced in both senescent HEI-OC1 cells and supernatant. Notably, the senescent HEI-OC1 supernatant stimulated BV2 cells CD74 expression increased. rCD74 significantly upregulated apoptosis-related genes expression levels in HEI-OC1 cells, while decreasing MIF levels. In the co-culture system of scrambled/CD74^-^BV2 cells and HEI-OC1 cells, CD74^-^ BV2 cells markedly reduced the apoptosis-related genes expression in senescent HEI-OC1 cells. MIF could improve the mitochondrial membrane potential of both groups of HEI-OC1 cells, and decreased the TUNEL positive cells significantly. Our findings using the HEI-OC1 cell model reveal that macrophages secrete CD74 into the microenvironment, where it interacts with MIF, reducing local MIF levels. This interaction weakens MIF’s protective effect on senescent HEI-OC1 cells, promoting apoptosis in these auditory cells. This suggests a potential mechanism whereby elevated CD74 in the aging cochlear microenvironment may contribute to the loss of hair cells *in vivo*. Targeting macrophage CD74 may offer therapeutic potential for preventing and treating age-related hearing loss.

## Graphical Abstract

**Figure:**
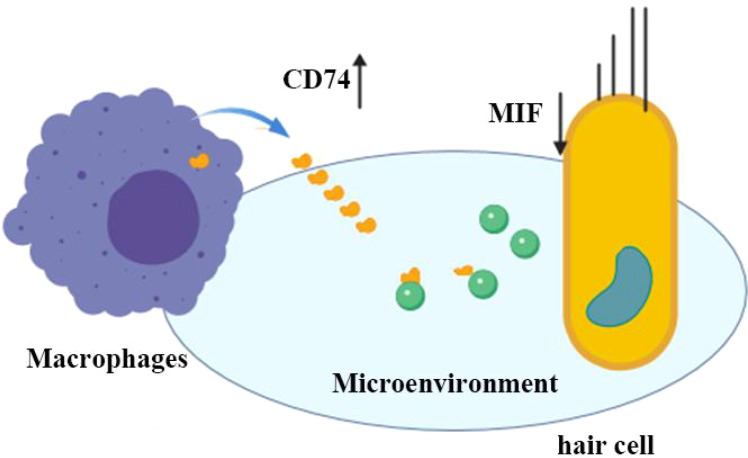

## Introduction

Age-related hearing loss, or presbycusis, is a global public health issue and one of the most common chronic conditions among the elderly, especially with the ongoing process of population aging. It is primarily characterized by reduced auditory sensitivity and impaired speech recognition, particularly in noisy environments. This significantly affects the quality of life of elderly individuals, potentially leading to social isolation, depression, and interactions with various other conditions, such as neurosis, cognitive decline, and dementia.

Chronic low-grade inflammation can occur in aging tissues and tends to worsen with age (1, 2). Recent studies suggest that inflammatory aging may trigger the onset and progression of presbycusis. Inflammatory responses in the cochlea involve the recruitment of circulating immune cells and the release of inflammation-related mediators, leading to persistent inflammation. Studies have shown that elevated levels of inflammatory markers in circulation are associated with age-related hearing loss (3–6). Macrophages are the main resident immune cells in the inner ear, playing roles in synapse repair and the clearance of cellular debris. Resident macrophages are derived from fetal liver and the yolk sac, specifically expressing CX3CR1 and CD163, and are distributed in the Rosenthal’s canal, spiral ganglion neurons (SGN), basilar membrane, spiral ligament, stria vascularis (7, 8). Moreover, macrophages in the inner ear change morphology and distribution in response to various stimuli, including aging.

Macrophage activation may contribute to sensory cell loss. Cochlear macrophages in the basilar membrane mainly aggregate on the scala tympani side and are the immune cells closest to hair cells spatially. They can actively sense changes in the status of sensory epithelial cells and respond accordingly (9). Degeneration of inner ear sensory cells leads to the activation of macrophages in the basilar membrane, accompanied by morphological changes in these macrophages. Compared to young cochleae, aged cochleae show increased numbers of macrophages around sensory epithelia in the middle and basal turns, with enlarged cell bodies, a granular appearance, and a rise in the number of amoeboid macrophages. These amoeboid macrophages are associated with significant outer hair cell loss in the cochlea and increased interactions with myelinating glial cells and auditory neurons, indicating more pronounced immune functions (10–12). Morphologically, macrophages in the aged cochlea are predominantly in an activated state. Activated macrophages can produce and release pro-inflammatory mediators, promoting inflammation and inducing cellular damage or apoptosis (13, 14). Studies have observed that macrophage activation precedes sensory cell loss, and cumulative, progressive degeneration and death of sensory cells can further lead to the activation of resident macrophages (11).

CD74 is a 33.5 kDa homotrimeric, non-polymorphic type II transmembrane glycoprotein (15, 16). Its primary biological function is to act as a chaperone for exogenous peptides and MHC class II molecules, participating in antigen presentation and interacting with the cytokine MIF. Recent research shows that the secreted form of CD74 is involved in inflammatory responses and apoptosis in the body (17). Assis et al (18) found that serum levels of soluble CD74 (sCD74) were significantly elevated in patients with autoimmune hepatitis and primary biliary cholangitis. Under IFN-γ stimulation, THP-1 macrophages can release CD74, which acts on MIF in the tumor microenvironment to inhibit tumor cell growth and induce apoptosis (19). Circulating CD74 in patients with autoimmune liver diseases and primary biliary cholangitis can serve as a receptor for MIF (18). Circulating CD74 can bind and neutralize MIF competitively, thereby affecting the biological function of MIF. Studies indicate that macrophage migration inhibitory factor (MIF) is widely expressed in the inner ear and plays a crucial role in maintaining inner ear development and normal hearing. MIF is highly expressed in various parts of the mouse inner ear, including the spiral ligament, stria vascularis, spiral limbus, spiral ganglion cells, and the organ of Corti (20). As a neurotrophic factor, MIF plays a vital role in the development of the vertebrate auditory system and is an important factor in maintaining normal cochlear function. MIF-knockout mice exhibit early hearing impairment, altered innervation of the organ of Corti, and a reduction in sensory hair cells (21–24). MIF also plays a significant role in the development of presbycusis. Kariya, S. et al. (25) found that MIF-deficient mice exhibited accelerated age-related hearing loss and morphological abnormalities of the inner ear. Compared with wild-type mice, they showed more pronounced high-frequency hearing loss and significant damage to outer hair cells at 9, 12, and 18 months of age, while inner hair cells were relatively well-preserved, and the number of spiral ganglion cells was reduced. Zhang et al. (26) found that knocking down MIF in the mouse cochlea exacerbated hearing loss while overexpressing MIF could partially improve hearing in aged mice.

In summary, MIF is an important factor in maintaining auditory function in the aging inner ear microenvironment. It remains unclear whether cochlear macrophages, as the major immune cell subset in the inner ear, can secrete CD74 and act on MIF to affect hair cell survival and the progression of presbycusis. In this study, we used the widely applied House Ear Institute-Organ of Corti (HEI-OC1) cell line exposed to D-gal as a model to simulate age-related hearing loss. We investigated, for the first time, the effects of macrophage-derived CD74 on the damage of aging HEI-OC1 cells and revealed that macrophage CD74 aggravates the apoptosis of aging HEI-OC1 cells by acting on MIF in the microenvironment.

## Materials and methods

### Cell culture and treatment

The HEI-OC1 cell line was donated by Professor Zheng Yiqing’s team from Sun Yat-sen Memorial Hospital of Sun Yat-sen University. The cells were cultured in high-glucose Dulbecco’s Modified Eagle Medium (DMEM) (Gibco, China) supplemented with 10% fetal bovine serum (Gibco, China), and maintained at 33°C in a 10% CO2 incubator. BV2 cells were cultured in high-glucose DMEM medium, supplemented with 10% FBS and 1% penicillin/streptomycin, and maintained at 37°C in a humidified incubator with 5% CO2.

### Flow cytometry

Cells were washed with PBS and digested with 0.25% EDTA-trypsin for 1 minute. Cells were centrifugation at 300g for 5 minutes. F4/80 and CD74 antibodies were prepared in a mixture using flow cytometry buffer, and the cells were stained at 4°C for 30 minutes, followed by washing with 1ml of flow cytometry buffer, and centrifuged at 300g for 5 minutes twice. The cells were resuspended in 1% paraformaldehyde, protected from light, stored at 4°C. Analysis was performed using CytoFlex cytometer.

### Cell viability assay

HEI-OC1 cells were seeded into a 96-well plate 5000 cells/well and incubated overnight at 33°C in a 10% CO2 incubator. Then cells were treated with varying concentrations of D-galactose for 48 hours. After treatment, the medium was replaced with a 10% Cell Counting Kit-8 (CCK-8) working solution and incubated for an additional 45 minutes, and the OD values at 450 nm were measured using a microplate reader. Cell viability was calculated.

### Cell apoptosis assay

Cell apoptosis was determined using the Annexin V-FITC apoptosis kit (C1062M, Beyotime, Shanghai, China). After treatment, the supernatant was collected in centrifuge tubes, and cells were harvested using EDTA-free trypsin, then combined with the supernatant in the same tubes. The cells were centrifuged at 300g for 5 minutes. A total of 3-5 × 10^5 cells were centrifuged at 300g and resuspended in 100 μL of Annexin V Binding Buffer with 2.5 μL of Annexin V and 2.5 μL of propidium iodide and incubated in the dark for 20 minutes. Subsequently, 400 μL of Annexin V Binding Buffer was added to each sample, which was analyzed using flow cytometry on a CytoFlex cytometer while kept on ice and protected from light.

### Enzyme-linked immunosorbent assay

Supernatant was collected and centrifuged at 3000 rpm for 15 minutes to remove cell debris and impurities after treated as required. ELISA was performed according to the manufacturer’s instructions. The OD values were measured at 450 nm, and a regression equation was generated using the OD values of the standards in Excel. The sample concentrations were calculated by inputting the sample OD values into the equation and multiplying by the dilution factor to obtain the final concentration.

### Quantitative real-time polymerase chain reaction

Total RNA was extracted from samples using the Trizol (Cat # 15596018, Invitrogen) and measured with a spectrophotometer, according to the manufacturer’s instructions. Reverse transcription was performed using the 5X PrimeScript RT Master Mix(Cat # RR036A, Takara) synthesize cDNA. q-PCR was performed on Applied Biosystems 7500 PCR system. The following primer pairs were used for detection:Actin, forward 5’- TTCAACACCCCAGCCATG-3’ and reverse 5’-CTCGTAGATGGGCACAGT-3’; Cd74, forward 5’- CATGGATGACCAACGCGAC-3’ and reverse 5’-TGT ACAGAGCTCCACGGCTG-3’; MIF, forward 5’-CCAGAACCGCAACTACAGTAAGC-3’ and reverse 5’-TTGGCAGCGTTCATGTCGTAATAG-3’; BCL2, forward 5’-GACTGAGTACCTGAACCGGC-3’ and reverse 5’-AGTTCCACAAAGGCATCCCAG; Bax, forward 5’-AAACTGGTGCTCAAGGCCC-3’ and reverse 5’-GGTCCCGAAGTAGGAGAGGA-3'. Actin was used as an internal control for normalization.

### Senescence-associated β-galactosidase activity assay

Cell senescence was assayed with the aid of a senescence-associated β-galactosidase (SA-β-gal) assay kit (C0602, Beyotime, Shanghai, China). Briefly, the cells grown in a 6-well plate were washed with PBS and fixed for 15 min by adding 1 ml β-galactosidase staining fixation solution. After fixation, the cells were washed with PBS and incubated overnight at 37 °C with the staining solution mix. SA-β-gal positive cells were stained blue-green under a light microscope.

### Detection of the mitochondrial membrane potential

Cell mitochondrial membrane potential was assessed according to the manufacturer’s instructions. After treatment, cells were digested with 0.25% trypsin and centrifuged at 1000 rpm for 5 minutes. 500 μL of JC-1 working solution was added, and the cells were incubated at 37°C for 30 minutes. The cells were then centrifuged at 300g for 5 minutes, the supernatant was discarded, and the cells were washed with pre-cooled 1X JC-1 Assay Buffer. The cells were resuspended in 300 μL of 1X JC-1 Assay Buffer and subsequently analyzed using the CytoFlex flow cytometer.

### TUNEL staining (terminal deoxynucleotidyl transferase-mediated dUTP-biotin nick end labeling)

Cells were fixed with 4% paraformaldehyde for 30 minutes after treatment. Samples were permeabilized with 0.2% Triton X-100 for 10 minutes, followed by three additional PBS washes. A 100 μL volume of TdT Equilibration Buffer was applied to the sample area marked with a hydrophobic barrier pen, and the samples were equilibrated at 37°C for 20 minutes. Then TUNEL working solution was added to the samples, and they were incubated in a humidified chamber at 37°C for 60 minutes, protected from light. Then cell nuclei were counterstained with DAPI for 5 minutes. The samples were mounted with an anti-fade mounting medium and observed under a fluorescence microscope for imaging.

### Lentiviral transduction of BV2 cells

BV2 cells were prepared and seeded in a 24-well plate. The growth and confluence of the cells were monitored. Lentiviral particles were thawed from -80°C and diluted into five virus concentration gradients (0, 2, 4, 8, 10 μL). The old culture medium was carefully removed, and media containing the virus was added along with 6 μg/mL polybrene. The mixture was gently mixed and incubated overnight at 37°C in a 5% CO2 incubator. After 12–16 hours, the virus-containing medium was discarded, and fresh medium with 5% FBS was added. The cells were then cultured and monitored until they reached approximately 80% confluence. Puromycin was added for selection, and the cells were further cultured with media changes performed daily. Once the cells reached a stable state, they were collected for qPCR analysis to assess the efficiency of knockdown. CD74-knockdown BV2 and scrambled BV2 cells were cultured in high-glucose DMEM supplemented with 10% fetal bovine serum, 4 μg/mL puromycin, and 1% penicillin/streptomycin.

### Co-culture system of BV2 and HEI-OC1 cells

HEI-OC1 cells were seeded at the bottom of a culture plate and cultured in DMEM supplemented with 10% heat-inactivated fetal bovine serum and 100 U/mL penicillin, and incubated at 33°C in a 10% CO2 incubator. After 24 hours, the medium was replaced with D-galactose-containing medium, and the cells were further cultured for 48 hours before co-culture. BV2 cells were seeded into the corresponding Transwell inserts and incubated at 37°C in a 5% CO2 incubator for 24 hours before use in the co-culture system. The Transwell inserts containing BV2 cells were then transferred into the culture plates with HEI-OC1 cells to establish a co-culture system with BV2 cells in the upper chamber and HEI-OC1 cells in the lower chamber.

### Western blot analysis

Samples were lysed and centrifuged at 12,000 r/min for 10 minutes at 4°C. Protein concentration in the supernatant was measured by BCA protein assay kit (Thermo Scientific, USA). The samples were then mixed with 5X loading buffer (Solarbio, China) and boiled at 100°C for 5 minutes. Proteins were separated by SDS-PAGE, transferred onto polyvinylidene difluoride (PVDF) membrane. The membrane was then incubated overnight at 4°C in specific primary antibody and HRP-conjugated secondary antibody for one hour in room temperature. Protein signals were detected using ECL (Millipore, USA) chemiluminescent substrate. Immunoblots were imaged and the relative signal intensity of the target proteins to the loading control was quantified and normalized using ImageJ software (NIH, USA).

### Auditory brainstem response

Four-week-old and ten-month-old C57BL6J mouse were obtained from the Laboratory Animal Center of Sun Yat-Sen university and Zhejiang Vital River Laboratory Animal Technology Co.,Ltd. The animal use was approved by the Institutional Animal Care and Use Committee (IACUC), Sun Yat-Sen University (SYSU-IACUC-2024-001166),and the Laboratory Animal Ethics Committee of the Sixth Affiliated Hospital of Sun Yat-sen University(IACUC-2024060301). ABR test was conducted in an electromagnetically shielded soundproof chamber. Hearing thresholds were measured using the Tucker-Davis Technologies System 3. The RP2.1 module generated digitized signals with the following parameters: 10 ms duration, 0.5 ms rise/fall time. The digitized signals were amplified and transmitted to a speaker, then delivered to the mouse’s external auditory canal via a connected tube. The responses were recorded using a cranial electrode placed on the mouse’s forehead, with the reference electrodes inserted subcutaneously behind the test ears, ensuring resistance was below 1000Ω. Hearing thresholds for pure tones at 8 kHz, 16 kHz, 24 kHz, and 32 kHz were tested. Each signal was repeated and averaged 500 times in SigGen. The test began at 90 dB SPL for each frequency, decreasing by 10 dB until no waveform could be elicited. If no response was observed, the intensity was increased by 5 dB. The threshold for each frequency was recorded as the lowest intensity at which a waveform could no longer be detected.

### Mouse cochlear preparation

Mice were sacrificed and decapitated after satisfactorily anesthetized by 4% inhaled isoflurane. A midline sagittal incision was made along the skull to remove the brain tissue, exposing the auditory bulla. The bullae were extracted and placed in pre-cooled PBS dish. Under a stereomicroscope, excess soft tissue was carefully removed. The cochlea was dissected by severing the connection between the cochlear and vestibular regions along the line between the round window and oval window, with the vestibular part discarded. The cochlea was then transferred into a 2 mL round-bottom Locksafe centrifuge tube containing a 7 mm stainless steel bead. After pre-cooling in liquid nitrogen, the cochlea was pulverized by Eppendorf tissue grinder at 50 Hz for 2 minutes.

### Bioinformatics analysis

Single-cell RNA sequencing data were obtained from the Genome Sequence Archive (GSA; project CRA004814), comprising cochlear tissues from C57BL/6J mice at ages 1, 2, 5, 12, and 15 months. Raw FASTQ files were processed using Cell Ranger with the mm10 reference genome for alignment, barcode/UMI counting, and generation of the feature-barcode matrix. Seurat was used for single-cell data analysis, including data integration, clustering, and dimensionality reduction analysis in R. Cell types were annotated automatically with the SingleR package (reference: MouseRNAseqData) and manually refined using canonical marker genes from the literature. Immune cell and sensory epithelial cell populations were subset and re-clustered independently for higher-resolution analysis. Differentially expressed genes (DEGs) for each cluster were identified using the FindAllMarkers function with thresholds of P.adj < 0.05 and |log2FC| > 1. Pathway enrichment analysis of DEGs was performed using the ClusterProfiler package for Kyoto Encyclopedia of Genes and Genomes (KEGG) terms.

To investigate potential interactions between macrophages and hair cells, receptor-ligand analysis was conducted. Expression matrices for the respective subpopulations were extracted and analyzed using the CommPath web tool (https://commpath.omic.tech/). Default parameters were applied to identify statistically significant receptor-ligand pairs and visualize the communication networks.

### Statistical analysis

Data are presented as mean ± SD. Statistical analyses were conducted using GraphPad Prism 9, with P < 0.05 considered statistically significant. Comparisons between two independent groups were made with unpaired t-tests. While, multi-group comparisons were conducted as follows: One-way ANOVA with Tukey’s multiple comparisons test was used to compare means across three or more groups under a single experimental condition. Two-way ANOVA with Sidak’s multiple comparisons test was applied to analyze data with two independent factors.

## Results

### Cochlear CD74 expression increases during aging

**Figure 1:**
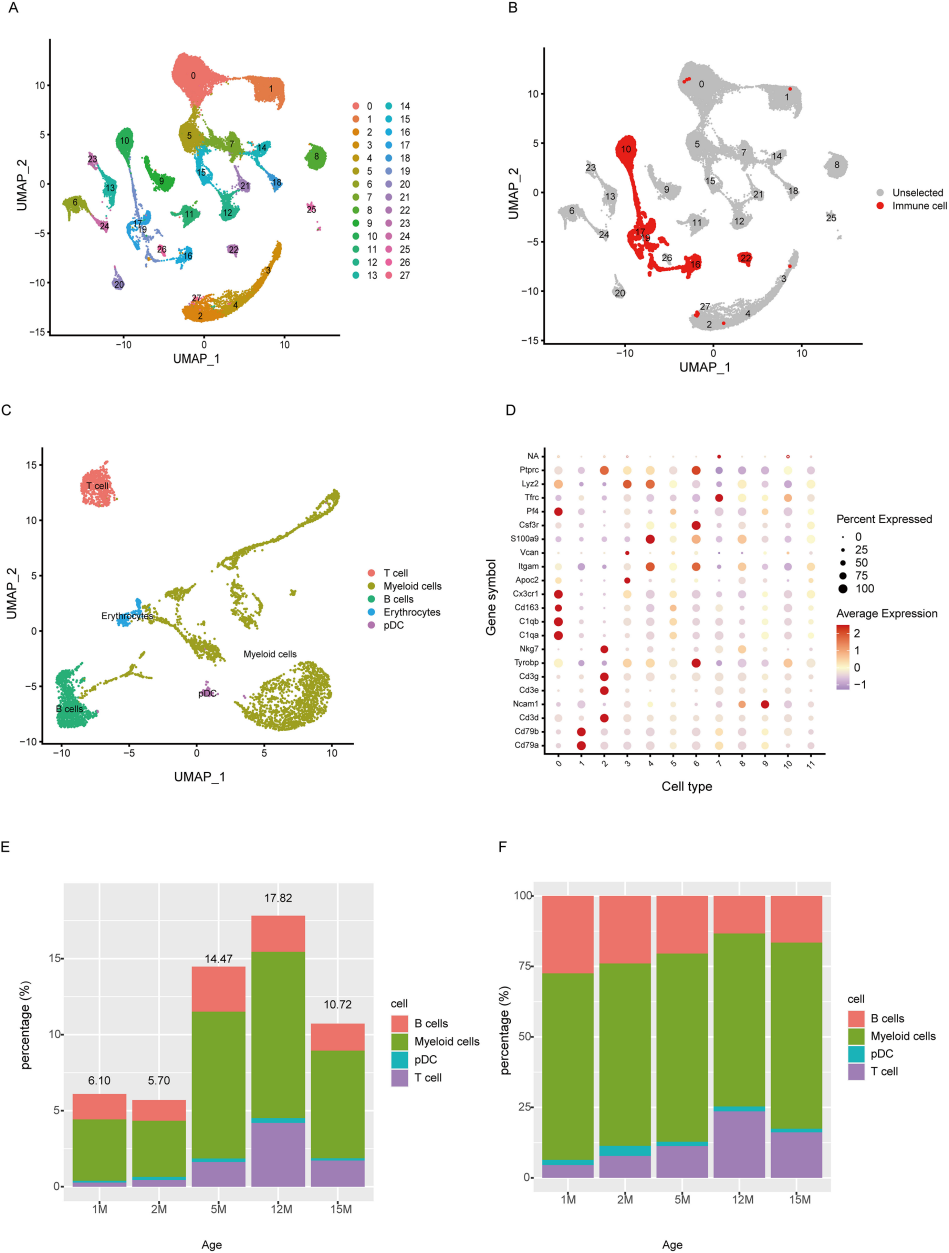
Immune cell subpopulations changes in the Cochlea at different ages. **(A)** Overall UMAP plot of cochlear samples, identifying cell subpopulations in the mouse cochlea through single-cell sequencing. **(B)** UMAP showing immune cell subpopulations (red). **(C)** UMAP of immune cell subpopulations. **(D)** Expression of marker genes identifying immune cell subpopulations. **(E)** Proportion of immune cells relative to the total number of cells in the sample. **(F)** The composition of immune cell subsets across different age groups.

C57BL/6J mice exhibit progressive hearing loss accompanied by cochlear and auditory cortex degeneration, making them a widely used animal model for age-related hearing loss (ARHL) (27). In this study, we obtained dataset CRA004814 from the Gene Sequence Archive (GSA) at the Chinese Academy of Sciences. This dataset comprises single-cell RNA sequencing data derived from the cochleae of C57BL/6J mice at 1, 2, 5, 12, and 15 months of age. Basic information about the data can be found in S1. We identified 28 cell subpopulations, according to integration and unsupervised clustering (**Figure 1A**). Based on SingleR automated cell annotation and further optimization of marker genes in the literature, we identified clusters 10, 16, 17, 19, and 22 as immune cell subpopulations (**Figure 1B**). T cells highly expressed CD3D, B cells expressed CD79A and CD79B, and macrophages expressed Cx3cr1. We extracted immune cell subpopulations for further analysis, focusing on changes at different ages.

When analyzing the proportion of immune cells relative to total cochlear cells, we found that immune cells made up a small proportion during cochlear development, comprising 6.10% and 5.70% at 1 and 2 months, respectively, showing a slight decrease. However, as maturation progressed to aging, the proportion of immune cells gradually increased, reaching 14.47% at 5 months, peaking at 17.82% in 12-month-old mice, and decreasing to 10.72% at 15 months (**Figure 1E**). Among all immune cells, myeloid cells had the highest proportion, followed by B cells, and T cells, while pDCs had a lower proportion (**Figure 1F**).

**Figure 2:**
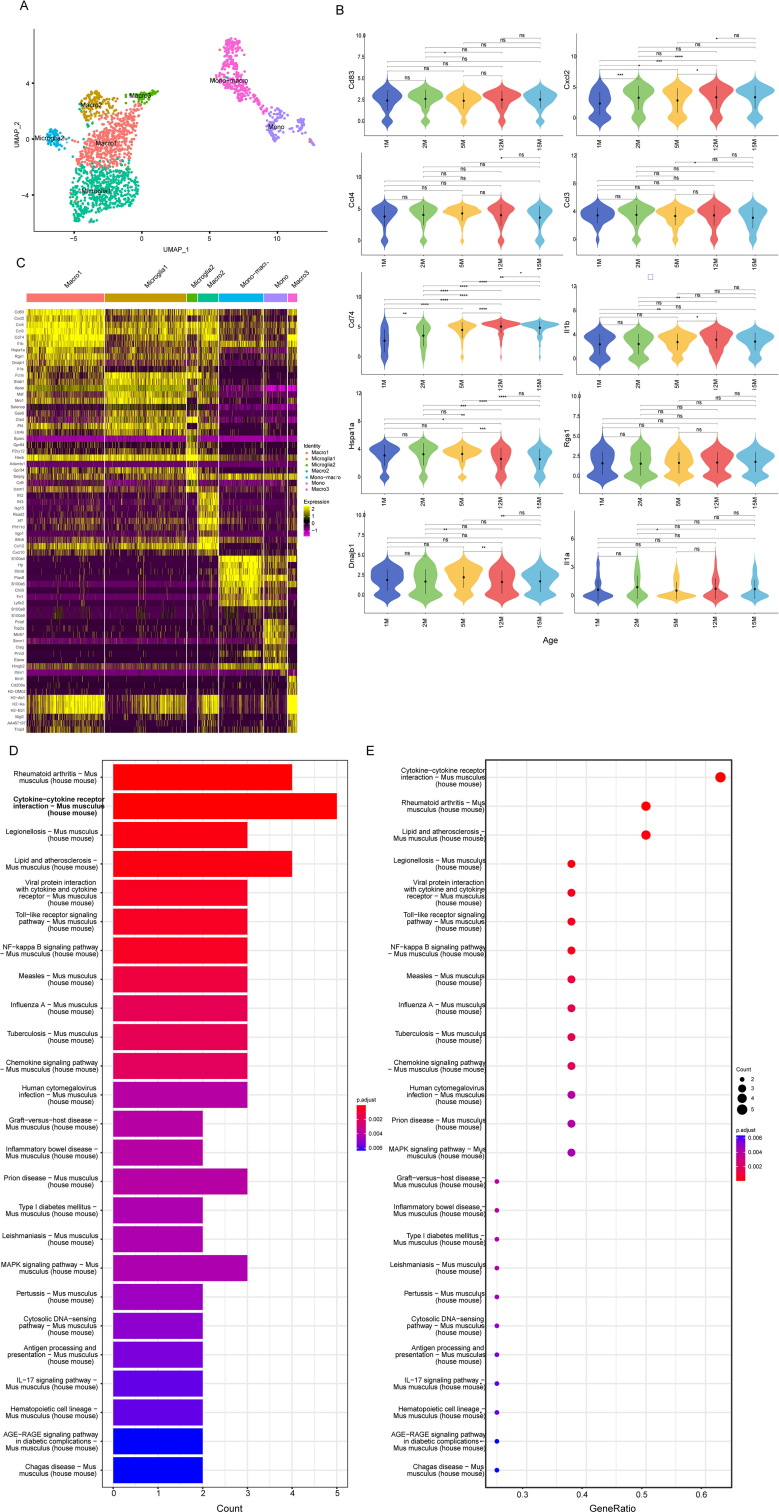
CD74 expression in macrophages gradually increases during cochlear aging. **(A)** UMAP plot of monocyte-macrophage subpopulations. **(B)** Top 10 differentially expressed genes Expression changes in macrophage cluster 1 across different ages (n=2, ns: p > 0.05,*p < 0.05,**p < 0.01,***p < 0.001,****p < 0.0001). **(C)** Heatmap of differentially expressed genes in various cell subpopulations. **(D)** KEGG Analysis of the Top 10 DEGs in Macrophage Subpopulations Bar plot; **(E)** Bubble chart.

To investigate the transcriptomic changes in cochlear macrophages with aging, we further analyzed the major macrophage subpopulation, cluster 1. The top differentially expressed genes in this subpopulation compared to other clusters were Cd83, Cxcl2, Ccl4, Ccl3, Cd74, Il1b, Hspa1a, Rgs1, Dnajb1, and Il1a, showing high expression levels (**Figure 2C**). We compared the expression changes of these top 10 genes across different ages in the cochlea, and the results showed that CD74 expression of macrophages increased progressively with age (**Figure 2B**). KEGG pathway analysis revealed enrichment in biological functions such as cytokine interactions, chemokine signaling pathways, antigen presentation, and inflammatory diseases, indicating that these macrophages may represent an activated subpopulation (**Figures 2D, E**).

**Figure 3:**
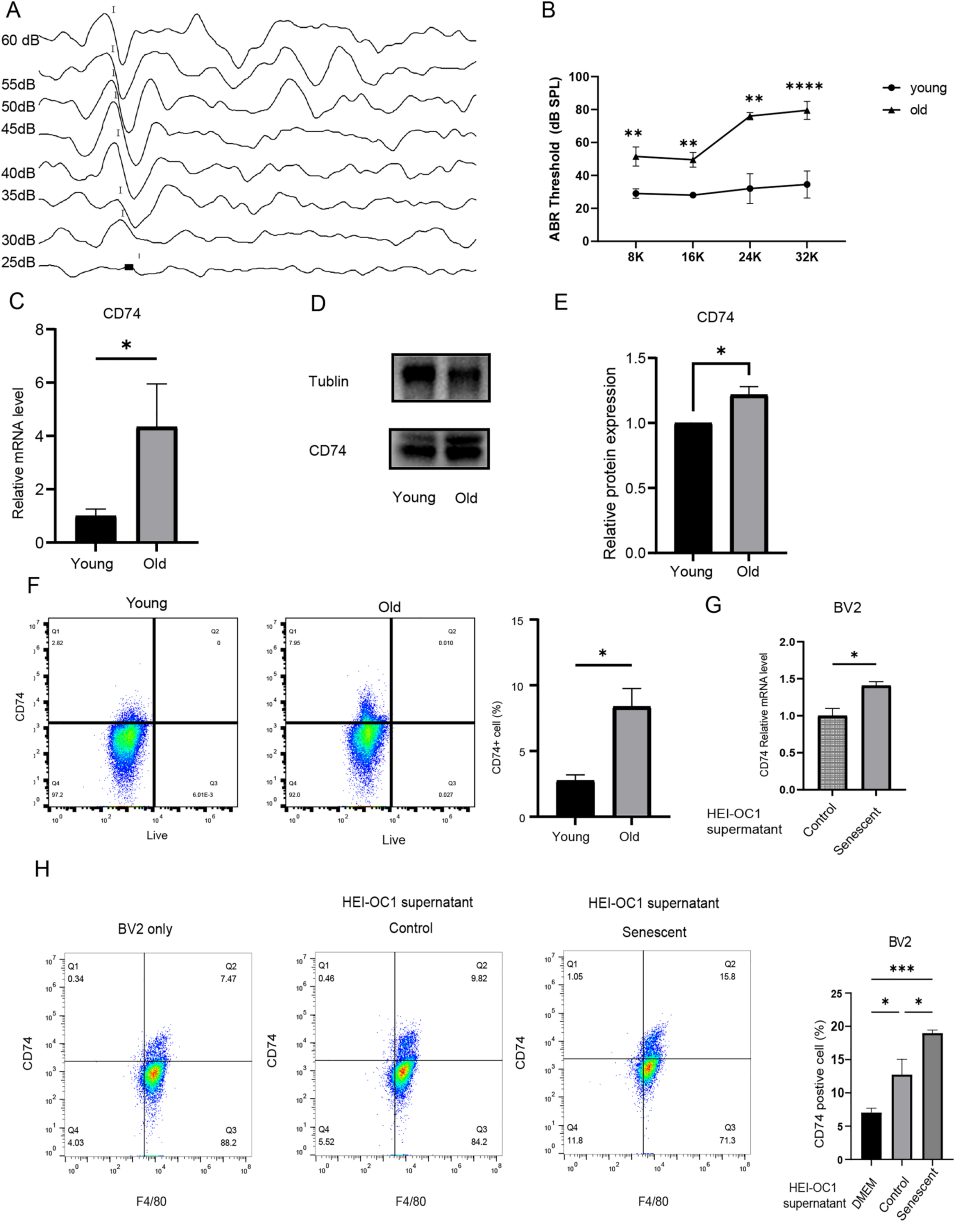
Increased CD74 expression in the cochlea of aged mice and BV2 cells. **(A)** auditory brainstem responses (ABR) at 24kHz pure tone stimulation in mice. (**B)** Comparative analysis of hearing thresholds at 8K, 16K, 24K, and 32K Hz frequencies of young and old mouse (n=5 mice, **P<0.01, ****P<0.0001, two-way ANOVA with Sidak’s multiple comparisons test ). **(C)** Increased CD74 mRNA expression levels of aged mouse cochlea (n=6 mice, *p < 0.05, t test). **(D, E)** immunoblot analysis of CD74 showing increased CD74 protein expression of aged cochlea (n=4 mice, *p < 0.05, t test). **(F)** FACS results showing the CD74-positive cells in the cochlea of young mice is significantly lower than in aged deaf mice (n=4 mice, *p < 0.05, t test). **(G)** Increased CD74 mRNA expression in BV2 cells stimulated by control and senescent HEI-OC1supernatant (n=3, *p < 0.05, one way ANOVA with Tukey’s multiple comparisons test). **(H)** FACS showing the CD74 expression levels in BV2 cells (n=3, *p < 0.05, ***p < 0.001, one way ANOVA with Tukey’s multiple comparisons test).

Hearing thresholds in young mice at 8K-16K-24K-32KHz are 29 ± 2.850 dB SPL, 28 ± 1.118 dB SPL, 32 ± 9.083 dB SPL, and 34.5 ± 8.178 dB SPL, respectively. In aged mice, hearing thresholds at 8K-16K-24K-32KHz are 51.50 ± 5.755 dB SPL, 49.5 ± 4.472 dB SPL, 76.00 ± 2.236 dB SPL, and 79.50 ± 5.420 dB SPL, respectively. Hearing thresholds at all frequencies were significantly elevated in aged deaf mice (p < 0.01) (**Figure 3B**).

Q-PCR showed that cochlea CD74 expression was significantly higher in the aged mice (p < 0.01) (**Figure 3C**). Western blot analysis revealed that the relative expression level of CD74 in the total cochlear protein of aged deaf mice was higher compared to young mice (p < 0.05) (**Figures 3D, E**). Additionally, flow cytometry analysis of single-cell suspensions from mouse cochlear tissue showed that CD74-positive cells in the cochlea of young mice were 2.753 ± 0.434%, significantly lower than the 8.385 ± 1.376% in aged deaf mice (p < 0.05). CD74 expression in cochlear tissue was significantly higher in aged mice compared to young mice (**Figure 3F**).

### Senescent HEI-OC1 stimulates BV2 cells CD74 expression increased

D-galactose is widely employed to induce senescence in both *in vitro* and *in vivo* models. In our study, a senescent HEI-OC1 cell model was established by D-galactose treatment (S2). To determine the optimal induction conditions, Cell viability was assessed using the CCK−8 assay at 48,72, and 96 h following treatment with 0, 45, or 60 mg/ml D-galactose. The results showed a dose-dependent significant reduction in cell viability with increasing D-galactose concentration. But the cell viability was similar across the three time points. 45 mg/ml D-galactose treatment for 48 hours is appropriate. Annexin V/PI staining also revealed apoptotic cells markedly increased, following D-galactose treatment. Q-PCR analysis showed a significant upregulation of the apoptosis-related gene ratio Bax/Bcl-2 in HEI-OC1 cells after D-galactose treatment (p < 0.05). Western blot results indicated a significant increase in proteins expression ratio Bax/Bcl-2. Therefore, the HEI-OC1 cells were treated with 45mg/ml D-galactose for 48 hours to establish an senescent model for subsequent studies.

After co-culturing the senescent HEI-OC1 cells conditioned medium with BV2 cells for 24 hours, BV2 cells CD74 mRNA levels significantly increased (p < 0.05) (**Figure 3G**). Further flow cytometry analysis of BV2 cells co-cultured with the conditioned medium from senescent HEI-OC1 cells showed a significant increase in CD74 expression levels (p < 0.05) (**Figure 3H**).

### CD74 upregulated Bax/Bcl-2 in senescent HEI-OC1 cells

**Figure 4:**
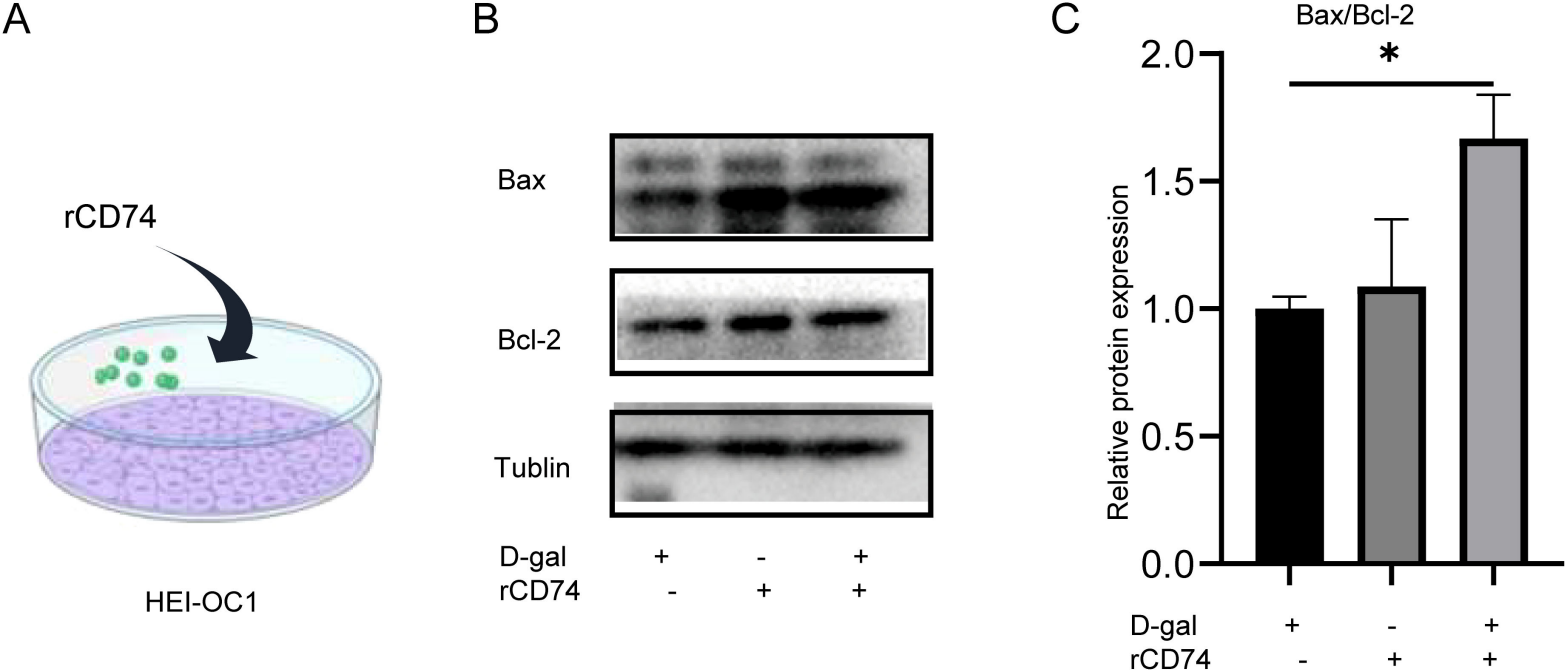
HEI-OC1 cells bax/bcl-2 expression levels after rCD74 treatment. **(A)** Schematic diagram of rCD74 treatment in HEI-OC1. **(B, C)** Western blot showing the expression of apoptosis-related proteins (n=4, *p < 0.05,one way ANOVA with Tukey’s multiple comparisons test).

We further investigated the effect on senescent HEI-OC1 cells after rCD74(recombinant CD74, rCD74) protein treatment (**Figure 4**). We examined the expression balance of the pro-apoptotic protein BAX and the anti-apoptotic protein BCL-2. The Bax/Bcl-2 ratio is a widely used molecular indicator of cellular commitment to apoptosis. The results showed that rCD74 significantly increased apoptosis-related protein Bax/Bcl-2 (p < 0.05) (**Figures 4B, C**). These data indicated that CD74 could exacerbate apoptosis in senescent HEI-OC1 cells, might play an essential role in senescent HEI-OC1 cell apoptosis.

**Figure 5:**
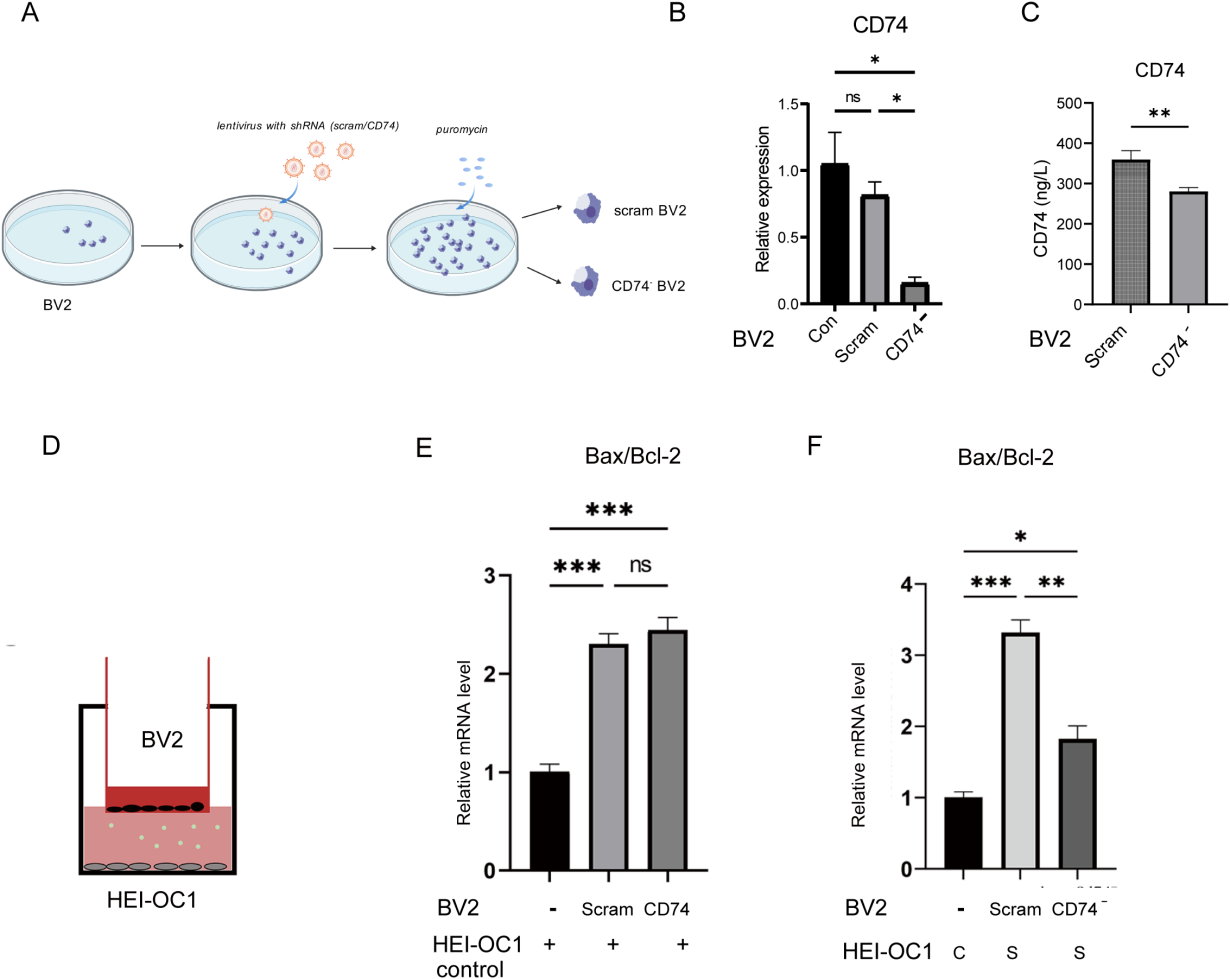
Knockdown BV2 cells CD74 reduced senescent HEI-OC1 cells apoptosis in co-culture system. **(A)** Schematic diagram showing the establishment of CD74 knockdown BV2 cells via lentiviral transduction. **(B)** CD74 mRNA expression levels changes in BV2 cells (n=3, ns: p > 0.05,*p < 0.05,one way ANOVA with Tukey’s multiple comparisons test ). **(C)** BV2 supernatant CD74 levels Changes (n=8, **p < 0.01, t test). **(D)** Schematic diagram of the transwell co-culture system of BV2 and HEI-OC1 cells, **(E)** BV2 cells increase the mRNA expression levels of apoptosis-related genes Bax/Bcl-2 in normal HEI-OC1 cells (n=3,ns: p > 0.05, ***p < 0.001,one way ANOVA with Tukey’s multiple comparisons test); **(F)** Knockdown BV2 cells CD74 expression reduced the mRNA expression levels of apoptosis-related genes Bax/Bcl-2 in senescent HEI-OC1 cells (n=3, ns: p > 0.05,*p < 0.05, **p < 0.01, ***p < 0.001, one way ANOVA with Tukey’s multiple comparisons test).

Could macrophage CD74 expression intervention mitigate HEI-OC1 cell apoptosis and protect senescent HEI-OC1 cells? Based on this hypothesis, we designed and constructed a lentivirus carrying a CD74 shRNA to knock down BV2 cells CD74 expression. Cells were transfected with the scrambled control shRNA are referred to as ‘scrambled BV2’ throughout this study. (**Figures 5A, B**). CD74 in the culture supernatant of CD74^-^ BV2 cells was significantly lower than that of scram BV2 (**Figure 5C**). We used BV2 cells as a macrophage model and established a transwell co-culture model with HEI-OC1 cells to simulate an inflammatory damage environment for hair cells *in vitro*. By using scrambled BV2 cells and CD74^-^BV2 cells, we created high and low sCD74 culture environments to act on senescent HEI-OC1 cells (**Figure 5D**).

When co-cultured with senescent HEI-OC1 cells in a lower CD74 environment, the ratio of apoptosis-related gene Bax/Bcl-2 in HEI-OC1 cells was significantly lower compared to co-culturing in a higher CD74 environment, indicating that a low CD74 environment can reduce senescent HEI-OC1 cells apoptosis. Macrophages regulated the senescent HEI-OC1 cells apoptosis by releasing endogenous CD74, and macrophage CD74 expression knockdown could decrease apoptosis in senescent HEI-OC1 cells (**Figure 5F**).

### Macrophage CD74 targeted inner ear MIF

**Figure 6:**
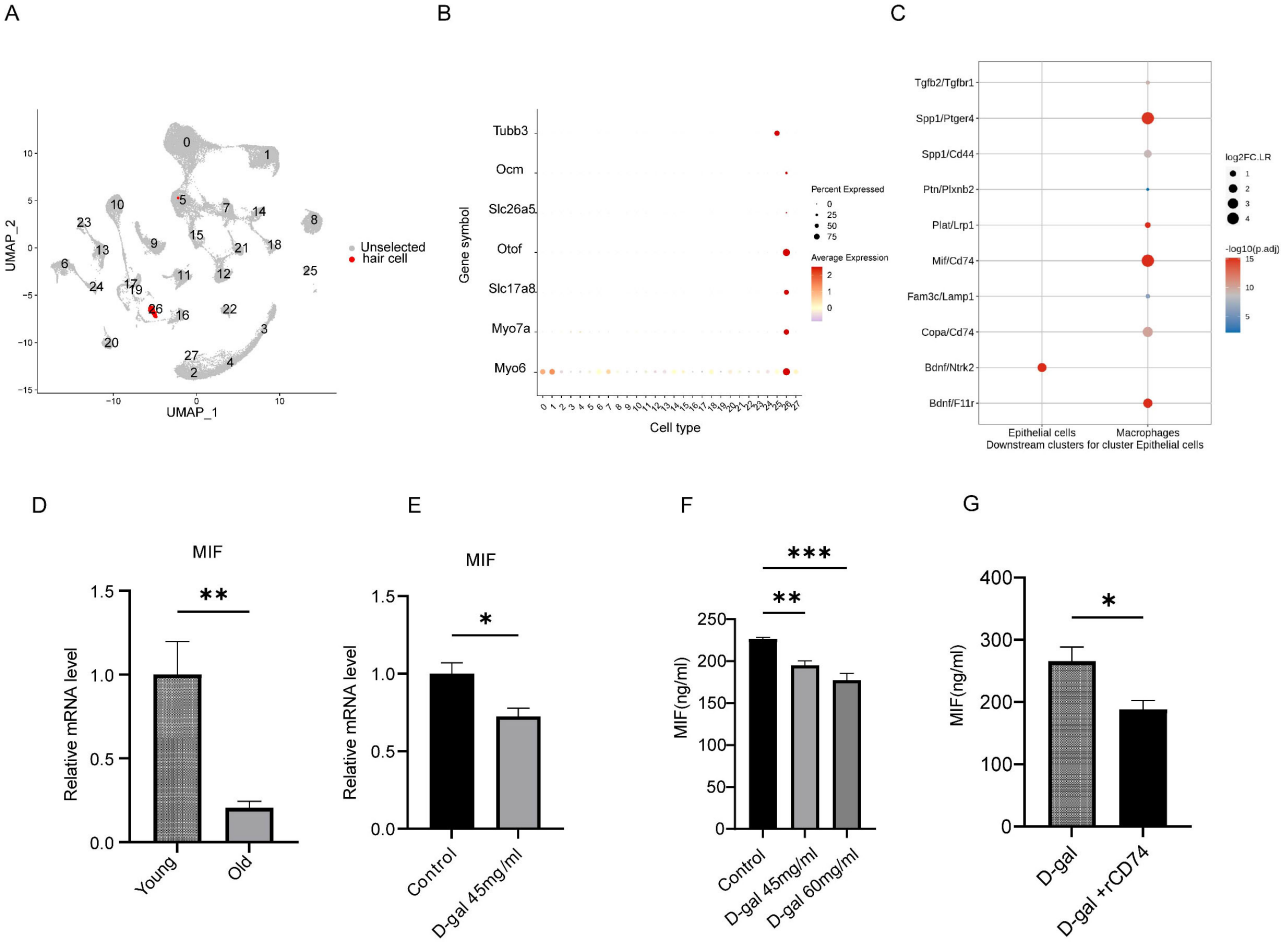
Decreased MIF expression in aged cochlea and senescent HEI-OC1 cells. **(A)** UMAP showing hair cells subpopulations in aged cochlea (in red). **(B)** Bubble plot displaying the expression of hair cell subpopulation marker genes (cluster26) in aged cochlea. **(C)** Top 10 receptor-ligand pairs of interaction between hair cells and macrophages. **(D)** Decreased MIF expression in aged cochlea (n=6 mice, **p<0.01, t test ). **(E)** Decreased MIF expression in senescent HEI-OC1 cells (n=6, *p<0.05, t test). **(F)** Decreased MIF levels in senescent HEI-OC1 cells supernatant (n=5, **p<0.01, ***p < 0.001, one way ANOVA with Tukey’s multiple comparisons test). **(G)** rCD74 reduced MIF levels of senescent HEI-OC1 cells supernatant (n=3 *p < 0.05, t test).

To identify the target of interaction between cochlear hair cells and macrophages, we extracted the inner ear sensory epithelial expression matrix for specific marker genes (Myo6, Slc26a5, Ocm, Slc17a8, Otof, Slc7a14, Tubb3) from the mouse cochlea single-cell data in the first part (**Figures 6A, B**). Cell communication analysis was performed on the expression matrices of auditory hair cells and macrophage subpopulations. This analysis revealed strong receptor-ligand interactions between MIF and CD74 between the two cell populations (**Figure 6C**).

MIF mRNA expression levels significantly decreased in the cochlea of aged mice (p < 0.01) (**Figure 6D**). Similarly, the MIF mRNA expression level in senescent HEI-OC1 cells was also significantly decreased (p < 0.05) (**Figure 6E**). ELISA measurements of MIF levels in the conditioned medium from D-galactose (0, 45 mg/ml, 60 mg/ml) treated cultures showed that MIF levels were 226.3 ± 1.979 ng/ml, 195.1 ± 5.146 ng/ml, and 177.3 ± 8.472 ng/ml, respectively. The MIF levels in the senescent HEI-OC1 conditioned medium were lower compared to the normal control group (p < 0.05) and decreased in a dose-dependent manner (**Figure 6F**).

Current data indicate that aging leads to decreased MIF expression in the cochlea and senescent HEI-OC1 cells, with MIF levels in the microenvironment also declining. To explore the effect of CD74 on MIF levels in the microenvironment, we added recombinant CD74 protein to the conditioned medium of senescent HEI-OC1 model cultures. Two treatment groups were conducted (D-galactose senescent model group, D-galactose senescent model + rCD74 group) and co-cultured for 48 hours, with MIF levels in the conditioned medium measured. Results showed that the MIF levels in the senescent HEI-OC1 cell culture environment further significantly decreased after treatment with rCD74 protein (p < 0.05) (**Figure 6G**). The data showed that rCD74 reduced MIF levels in senescent HEI-OC1 culture environments.

### MIF reduced apoptosis of senescent HEI-OC1 cells

**Figure 7:**
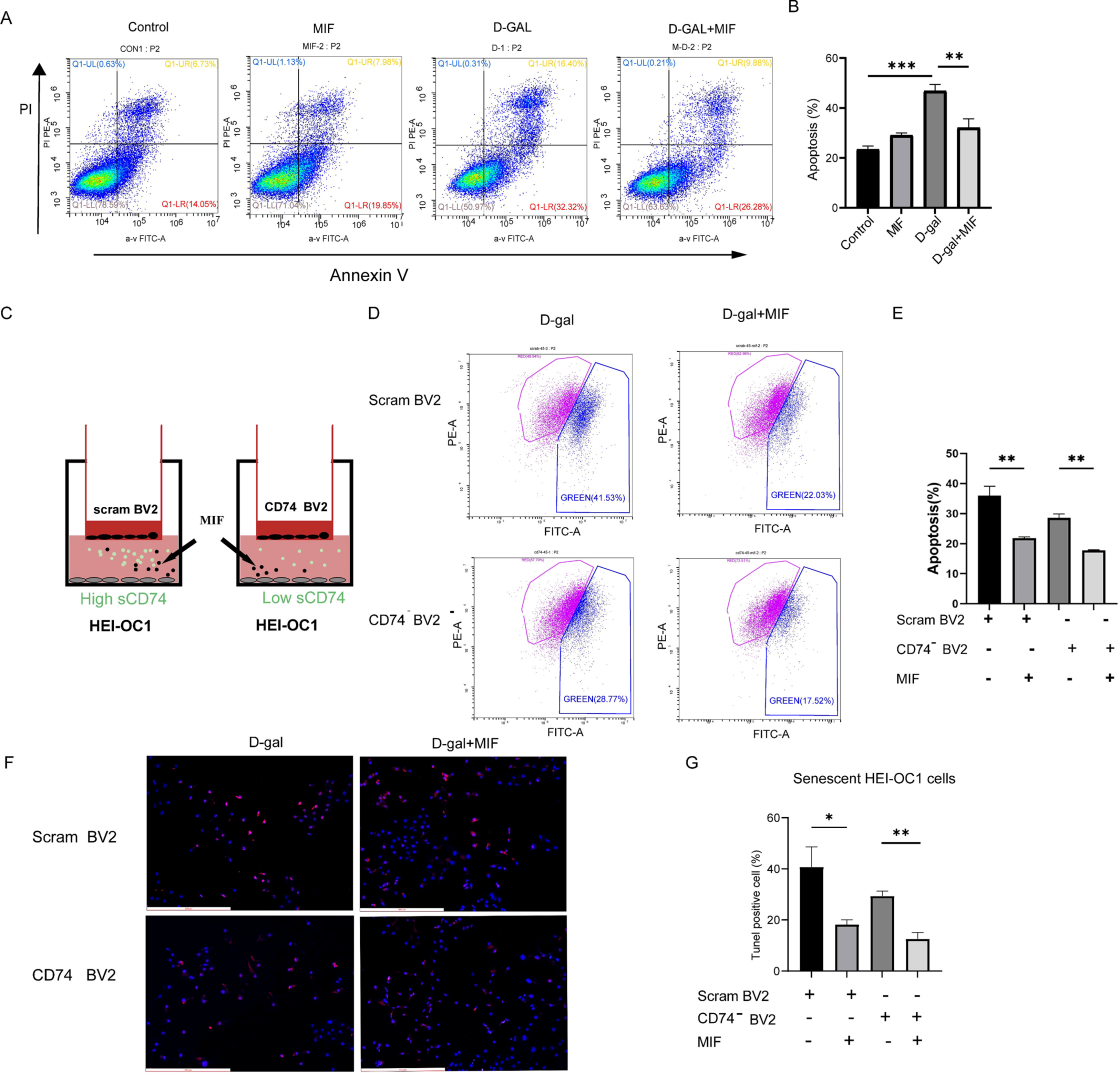
MIF reduced senescent HEI-OC1 cells apoptosis. **(A)** MIF reduced senescent HEI-OC1 cells apoptosis detected by Annexin V/PI flow cytometry. **(B)** Statistical analysis of the percentage of apoptotic cells (n=3,**p < 0.01, ***p < 0.001,one way ANOVA with Tukey’s multiple comparisons test). **(C)** Schematic diagram of MIF treatment in co-culture systems. **(D)** MIF reduced apoptosis in senescent HEI-OC1 cells in co-culture systems by JC-1 flow cytometry. **(E)** Statistical analysis of the percentage of apoptotic cells (n=3,**p < 0.01, one way ANOVA with Tukey’s multiple comparisons test). **(F)** TUNEL staining immunofluorescence images (10X, scale bar: 500 µm). **(G)** Statistical analysis of the proportion of TUNEL positive cells (n=3, *p < 0.05, **p < 0.01, one way ANOVA with Tukey’s multiple comparisons test).

To determine the effect of MIF on HEI-OC1 cells, recombinant MIF protein was added to the culture environment. Four groups were performed (control, MIF, D-gal, D-gal + MIF). Annexin V/PI results showed that MIF had no significant effect on apoptosis in control HEI-OC1 cells (p > 0.05). However, MIF treatment significantly reduced the apoptosis level in senescent HEI-OC1 cells (p<0.01) (**Figures 7A, B**).

To further explore the effect of MIF on co-culture environments on aging HEI-OC1 cells, we added 200 ng/ml MIF to the scram/CD74-BV2 & senescent HEI-OC1 co-culture system. Four groups were set up (scram BV2 & HEI-OC1, scram BV2 & HEI-OC1 + MIF, CD74^-^ BV2 & HEI-OC1, CD74^-^ BV2 & HEI-OC1 + MIF) and co-cultured for 24 hours. We then assessed mitochondrial membrane potential and the percentage of TUNEL-positive cells in senescent HEI-OC1 cells.

Mitochondrial membrane potential was measured by the JC-1 test kit. The PE channel detects JC-1 aggregates, while the FITC channel detects JC-1 monomers. Senescent HEI-OC1 cells co-cultured with CD74^-^BV2 exhibited lower green fluorescence compared to those co-cultured with scram BV2, indicating higher mitochondrial membrane potential. After MIF treatment, the proportion of green fluorescence in senescent HEI-OC1 cells was significantly reduced in both groups, improving mitochondrial membrane potential and significantly reducing cell apoptosis (p < 0.05) (**Figures 7D, E**). TUNEL staining results showed that MIF significantly reduced the percentage of TUNEL-positive cells in the CD74^-^ BV2/scram BV2 & senescent HEI-OC1 co-culture system (p < 0.05). Additionally, the proportion of TUNEL-positive cells was slightly lower in CD74^-^ BV2 co-cultured with senescent HEI-OC1 cells compared to scram BV2 co-cultured cells (**Figures 7F, G**).

## Discussions

This study reveals that the expression of CD74 molecules in cochlear macrophages gradually increases with cochlear aging, as shown by single-cell transcriptomic profiles. Our main findings include: (1) The expression of CD74 molecules increases progressively with cochlear aging, leading to inflammation in the cochlea of aged mice and a decrease in cochlear MIF expression. (2) Knockdown of macrophage CD74 reduces apoptosis of senescent HEI-OC1 cells. (3) Macrophages secrete CD74, which interacts with MIF in the inner ear, diminishing MIF’s protective effect on aging cochlear hair cells and thereby exacerbating hair cell apoptosis.

The cochlea is the sensory organ for hearing. Chronic stress related to aging can cause inflammation in the cochlea, a natural defense response. This ongoing inflammation is a key factor in age-related hearing loss, as indicated by increased levels of blood leukocytes and other markers in older adults. Additionally, the immune status of the cochlea is linked to age-related hearing loss (5, 6, 11, 28).

Our study analyzed single-cell sequencing data from the cochleae of aging mice and found that the proportion of immune cells, including myeloid cells, T cells, and B cells, increased with aging, with macrophages being the predominant immune population. This aligns with previous research (29). As age-related hearing loss progresses, cochlear macrophages become more numerous and enriched. These resident macrophages play a key role in immune surveillance, clearing debris from cochlear cells, and participating in inflammatory responses, all essential for tissue repair and homeostasis (30). Macrophages can phagocytize apoptotic cell fragments, including injured cochlear hair cells. However, the specific changes and mechanisms of macrophages in this process remain unclear. Our analysis of single-cell data from macrophage subsets across different ages identified a subset of macrophages marked by genes such as Cd83, Cxcl2, Ccl4, Ccl3, Cd74, Il1b, Hspa1a, Rgs1, Dnajb1, and Il1a. These macrophages represent the main population in the cochlea and are mainly involved in inflammatory diseases, cytokine interactions, antigen presentation, and chemokine signaling, indicating an activation state related to inflammation and antigen presentation. Our results confirmed the presence of cochlear microinflammation from a transcriptomic perspective. Comparing the expression levels of these differential markers across ages, we observed that not only does the number of macrophages increase with aging, but their transcriptomic profiles also change accordingly. Notably, CD74 expression increases progressively with age.

CD74 is a type II transmembrane protein with several established biological functions. It acts as a chaperone and transport auxiliary factor for major histocompatibility complex (MHC) class II molecules, participating in antigen presentation (31–33). Additionally, CD74, as a receptor for MIF, mediates intracellular signal transduction (34). MIF-CD74 signaling axis plays a crucial role in initiating oncogenic signaling pathways and inflammatory responses (35, 36). Recent studies have explored the biological functions and clinical significance of soluble CD74. For instance, Assis et al (18) demonstrated that circulating sCD74 and MIF profiles help differentiate between primary biliary cirrhosis and acute autoimmune hepatitis. Wu et al (17) found that soluble CD74 is released by alveolar macrophages in acute lung injury models. Studies showed that sCD74 was released by proteolytic cleavage and alternative splicing (18, 37, 38). However, there are no studies specifically addressing CD74 expression in macrophages related to age-related hearing loss.

Combining single-cell sequencing results from cochleae of mice at different ages with subsequent validation experiments of CD74 expression in aged mice, we clarified that in the C57BL/6 mouse model of age-related hearing loss, CD74 levels in cochlear macrophages increase with age. Literature reports show that after intraperitoneal injection of lipopolysaccharides in BALB/c mice, CD74-positive cells are observed in infiltrating inflammatory cells, middle ear mucosa, and inner ear, suggesting that cochlear inflammation stimulates increased CD74 expression (20). Acute inflammation induces elevated CD74 expression in cochlear and lung macrophages, indicating that increased CD74 expression reflects macrophage activation. In age-related hearing loss cochleae, CD74+CD14+ macrophages accumulation was found in the basilar membrane (39). Our study found that during gradual aging, CD74 expression in cochlear macrophages also increases slowly, suggesting a gradual activation of macrophages as cochlear aging progresses. This may be related to increased metabolic products in the aging cochlea stimulating macrophages. Elevated CD74 expression may be linked to the progression of age-related hearing loss and plays a crucial role in cochlear aging.

Hair cell damage is a critical pathological basis for age-related hearing loss. We found that metabolites from senescent HEI-OC1 cells can upregulate CD74 expression in macrophages. In the aging process, sensory cells of the mammalian inner ear, including spiral ligament cells, spiral ganglion cells, and the stria vascularis, undergo gradual degeneration (40, 41). Among these, outer hair cells are most affected, with damage starting at the apex and base of the cochlea and spreading throughout the organ of Corti, leading to hearing loss (40, 42). The interactions between aging hair cells and inner ear macrophages have been a major research focus, as it is widely accepted that damage to hair cells and cellular debris can trigger sterile micro-inflammation in the inner ear.

In this study, we stimulated BV2 cells with conditioned media from senescent HEI-OC1 cells and observed a significant increase in CD74 expression. Recent research on neurodegenerative diseases indicates that disease-associated and aging microglia share partially overlapping transcriptomic profiles, both showing upregulation of CD74 (43, 44). Additionally, a distinct type of highly activated microglia (HAM) expressing Lpl, Lgals3, Cst7, and CD74 has been identified in aging mice (44). Wu et al (17) found that CD74 is present in lung macrophages, with increased soluble CD74 (sCD74) levels in bronchoalveolar lavage fluid and serum during acute lung injury, correlating with lung permeability and inflammation. These findings suggest that sCD74 production is linked to inflammation and macrophage activation. Therefore, we propose that the rise in macrophage CD74 is not only associated with aging but also closely linked to macrophage activation by metabolites from aging or apoptotic hair cells.

Our findings indicate that macrophage CD74 can induce apoptosis in senescent HEI-OC1 cells. Despite the anatomical isolation of inner ear hair cells within the cochlear duct, we hypothesize that activated inner ear macrophages mediate hair cell damage through the secretion of CD74. Recent literature has elucidated CD74’s role in regulating cell apoptosis and survival, linking it to various pathological conditions, including acute respiratory distress syndrome, liver disease, and melanoma (17, 45) (19). These studies suggest that local tissue macrophages may release CD74 in pathological states, exerting corresponding biological activities and thereby affecting the survival and apoptotic responses of adjacent tissue cells.

The CD74 on the surface of macrophages also has certain effects. However, due to the rapid internalization of the CD74 on the cell membrane, it stays on the cell membrane for a short time. We consider the role of membrane CD74 in this co-culture model to be relatively minor. Additionally, while macrophages adjacent to the basement membrane can directly impair hair cells, a larger population of macrophages resides in other inner ear structures. Their effects on hair cells may have a greater impact through secretion into the surrounding tissue and lymphatic fluid. Based on this, our research focuses more on the secretion effect.

Cell apoptosis is vital for maintaining homeostasis; however, inner ear hair cells are terminally differentiated and cannot regenerate, leading to irreversible hearing loss upon apoptosis. Therefore, preventing hair cell apoptosis is crucial for preserving normal hearing. The endogenous apoptosis pathway, primarily initiated by mitochondrial signals, is the main mechanism for hair cell loss in age-related hearing loss. Notably, deletion of the mitochondrial pro-apoptotic gene BAK can help prevent this condition (46, 47).

Knocking down macrophage CD74 reduced apoptosis in senescent HEI-OC1 cells by decreasing CD74 levels in the microenvironment. Co-culturing CD74^-^ BV2 cells with senescent HEI-OC1 cells significantly reduced the Bax/Bcl-2 ratio in HEI-OC1 cells compared to scram BV2, which created a higher CD74 environment. This revealed that reduced CD74 levels in the microenvironment mitigate apoptosis in senescent HEI-OC1 cells within the co-culture system. We also observed that co-culturing with BV2 cells increases the apoptosis of HEI-OC1 cells indicating that macrophages may be stimulated by HEI-OC1 cells metabolites to produce inflammatory factors and influence HEI-OC1 cells apoptosis. Notably, knocking down CD74 did not reduce the normal HEI-OC1 cell apoptosis. We speculate that, compared to the senescent HEI-OC1 cells, normal HEI-OC1 cells are more robust and have a greater tolerance to stress, but the exact mechanisms remain unclear.

In this study, we analyzed single-cell sequencing data from the cochlea and identified a strong interaction between the MIF/CD74 receptor-ligand in subpopulations of sensory epithelial cells and macrophages in the inner ear, suggesting that inner ear MIF is a target for macrophage CD74. We observed a significant decrease of MIF in age-related hearing loss cochlea, consistent with previous reports (26, 48). Currently, there are no studies reporting changes about MIF levels in cells or culture supernatants of *in vitro* senescent HEI-OC1 models. Our study found that MIF expression was significantly decreased in a senescent HEI-OC1 cell model induced by D-galactose, consistent with the trend observed in animal models. MIF can be secreted by various cell types, including epithelial and immune cells, acting through autocrine and paracrine mechanisms. Zhu et al. (49) reported that HEI-OC1 cells secrete MIF in response to acute stress from oxygen-glucose deprivation, with MIF supplementation improving cell survival. However, no studies have investigated changes in MIF secretion by senescent HEI-OC1 cells. Our findings indicate that MIF levels in the culture environment decreased as HEI-OC1 cells aging, suggesting that reduced MIF may contribute to HEI-OC1 cells apoptosis. Furthermore, rCD74 led to an additional decrease in MIF levels in the senescent HEI-OC1 cells culture environment, accompanied by increased apoptosis. This highlighted the interaction between CD74 and MIF in the microenvironment.

MIF is a cytokine that serves as both a pro-inflammatory and neurotrophic factor, essential for the auditory system and normal cochlear function in vertebrates (22–24). Our study reveals that MIF acts as a “nutrient factor”. Supplementation with MIF significantly decreased apoptosis in senescent HEI-OC1 cells. Kariya et al. (50) demonstrated that MIF deficiency impairs recovery from auditory hair cell damage, leading to long-term hearing loss after noise exposure. In MIF-deficient mice, outer hair cells were significantly lost, while inner hair cells remained relatively intact, suggesting that outer hair cells are more sensitive to MIF loss, consistent with mechanisms underlying age-related hearing loss.

Zhang et al. showed that overexpressing inner ear MIF improved hearing in aged mice, by reducing apoptosis in perivascular-resident macrophage-like melanocytes (PVM/Ms) in the stria vascularis (26, 48). Our findings indicate that MIF in the microenvironment is crucial for the survival of senescent HEI-OC1 cells and may represent an additional mechanism. As a “nutrient factor,” MIF gradually decreases with cochlear aging, making senescent hair cells more susceptible to apoptosis under various injuries. Macrophages, as the primary immune cells in the inner ear, become activated in the aging cochlea, secreting CD74 into the microenvironment, which interacts with MIF and further reduces the MIF levels. This reduction of beneficial nutrient factors exacerbates hair cell apoptosis. Our study provides a new perspective on how inner ear immunity affects hair cell survival.

## Limitations

Although we adopted the D-galactose-induced senescent model for HEI-OC1 cells, which is acceptable to most researchers, the model only approximates some aspects of age-related cochlear degeneration and is influenced by various systemic and local factors. It cannot fully represent the pathological physiological changes of hair cell aging. Besides, BV2, a microglial cell line, is frequently used as a proxy for macrophages in studies of cochlear macrophages. However, it may not be able to completely recapitulate the phenotype and signal transduction pathways of cochlear tissue macrophages. Moreover, the interaction between inner macrophages and hair cells may involve other pathways besides the ones we reported, such as resident tissue macrophages directly causing damage to hair cells (noise-induced hearing loss model) (7). These pathways may common drive the inner ear hair cells damage.

Macrophage-secreted CD74 is primarily released from the cell surface through selective RNA splicing and membrane protein hydrolysis to generate sCD74 (18, 37, 38). This process depends on a series of intracellular biological events following macrophage activation. Activated macrophages show increased surface expression of CD74, which is then rapidly internalized, and the changing membrane CD74 is hydrolyzed and shed from the cell membrane, forming soluble CD74 that enters the inner ear microenvironment to exert its biological functions. In single-cell RNA sequencing studies of the brains of aging mice, CD74 was identified as a differential marker for highly activated microglial cell populations, and MIF released by neurons can act through HAM’s CD74 receptor to promote the immune chemotactic activity of microglia, thereby triggering immune-inflammatory responses in the aging brain (44). This suggests a more complex and nuanced relationship between macrophages, CD74, and local microenvironment MIF, with more intricate mechanisms of interaction that require further exploration and research.

## Conclusion

With cochlear aging, the expression of CD74 in inner ear macrophages increases. Macrophages secrete CD74 into the inner ear microenvironment, where it interacts with MIF and reduces its local concentration. This interaction diminishes the protective effects of MIF on senescent hair cells, leading to increased apoptosis.

These findings reveal an interaction pathway between inner ear macrophages and hair cells, highlighting a crucial mechanism by which inner ear inflammation contributes to age-related hearing loss. This study provides valuable insights into the relationship between inner ear immunity and hair cell damage, suggesting that the macrophage CD74-MIF pathway may serve as a potential therapeutic target for mitigating inner ear inflammation and age-related hearing loss.

## Data Availability

The original contributions presented in the study are included in the article/**Supplementary Material**. Further inquiries can be directed to the corresponding authors.

## Glossary

ABR: Auditory Brainstem Response
ARHL: Age-Related Hearing Loss
CCK8: Cell Counting Kit-8
CD74: Leukocyte Differentiation Antigen 74
D-gal: D-Galactose
DMEM: Dulbecco’S Modified Eagle Medium
DEGs: Differential express Genes
ELISA: Enzyme-Linked Immunosorbent Assay
FBS: Fetal Bovine Serum
HEI-OC1: House Ear Institute-Organ Of Corti 1
MIF: Macrophage Migration Inhibitory Factor
OD: Optical Density
q-PCR: Quantitative Real-Time PCR
rCD74: Recombinant Cd74
ROS: Reactive Oxygen Species
sCD74: Soluble Cd74
TUNEL: Terminal Deoxynucleotidyl Transferase-Mediated Dutp-Biotin Nick End Labeling
GSA: Gene Sequence Archive
