# Supplementary material for: Cochlear macrophage CD74 enhances the apoptosis of senescent cochlear hair cells by down-regulating MIF
Source: Front Immunol. 2026 Mar 10;17:1751126. doi: 10.3389/fimmu.2026.1751126 (PMC13008737; doi:10.3389/fimmu.2026.1751126)
Supplement: Supplementary file 1 [file DataSheet1.pdf]

Supplementary materials

S1. Basic information of cochlea single cell sequencing data of different ages mouse.

sample	No	Measured data (G)	cell count	Median gene count	mean reads	total reads
S1F	CRR315530	16.09	6,219	989	8,625	53,643,130
S1M	CRR315531	22.60	5,463	1,247	13,790	75,338,118
S2F	CRR315532	33.31	7,775	1,238	14,279	111,023,443
S2M	CRR315533	18.84	5,601	1,091	11,210	62,787,865
S5F	CRR315534	42.76	6,519	1,423	21,865	142,542,813
S5M	CRR315535	45.19	8,043	1,280	18,726	150,620,322
S12F	CRR315536	18.70	3,163	1,372	19,703	62,323,554
S12M	CRR315537	17.70	3,914	1,270	15,076	59,007,799
S15F	CRR315538	24.38	3,959	1,496	20,528	81,272,525
S15M	CRR315539	26.63	6,607	1,263	13,437	88,781,992

S2. Establishment of an senescent model in the HEI-OC1 cell using D -galactose

HEI-OC1 cells were treated with different concentrations of D-galactose (0 mg/ml, 45 mg/ml, 60 mg/ml), and cell viability was measured at 48, 72, and 96 hours. The results showed that cell viability decreased in a dose-dependent manner following D-galactose treatment. At 48, 72, and 96 hours, the cell viability was similar across these time points (FigS2B). After 48 hours of treatment with 45 mg/ml D-galactose, cell viability decreased by nearly half, reaching 56.30 ± 1.301%. After treatment with 60 mg/ml D-galactose, cell viability was 30.78 ± 0.7619%, with statistically significant differences compared to the control group ($p < 0.05$) (FigS2C). Annexin V/PI revealed a significant apoptotic cells increase after D-galactose treatment ($p < 0.05$) (Figure s2D). Senescence-associated β-galactosidase staining revealed that HEI-OC1 cells exhibited a distinct light blue color after treatment with 45 mg/ml D-galactose. Following treatment with 75 mg/ml D-galactose, shrunken apoptotic cells were observed (Figure s2E). qPCR indicated a

significant upregulation in the ratio of the pro-apoptotic gene Bax to the anti-apoptotic gene Bcl-2 ($p < 0.05$) (Fig s2F), and Western blot analysis confirmed a significant increase in the Bax/Bcl-2 ratio after D-galactose treatment (Fig s2G, Fig s2H). Based on these findings, we selected 45 mg/ml D-galactose treatment for 48 hours to establish an aging model of HEI-OC1 cells for subsequent studies.

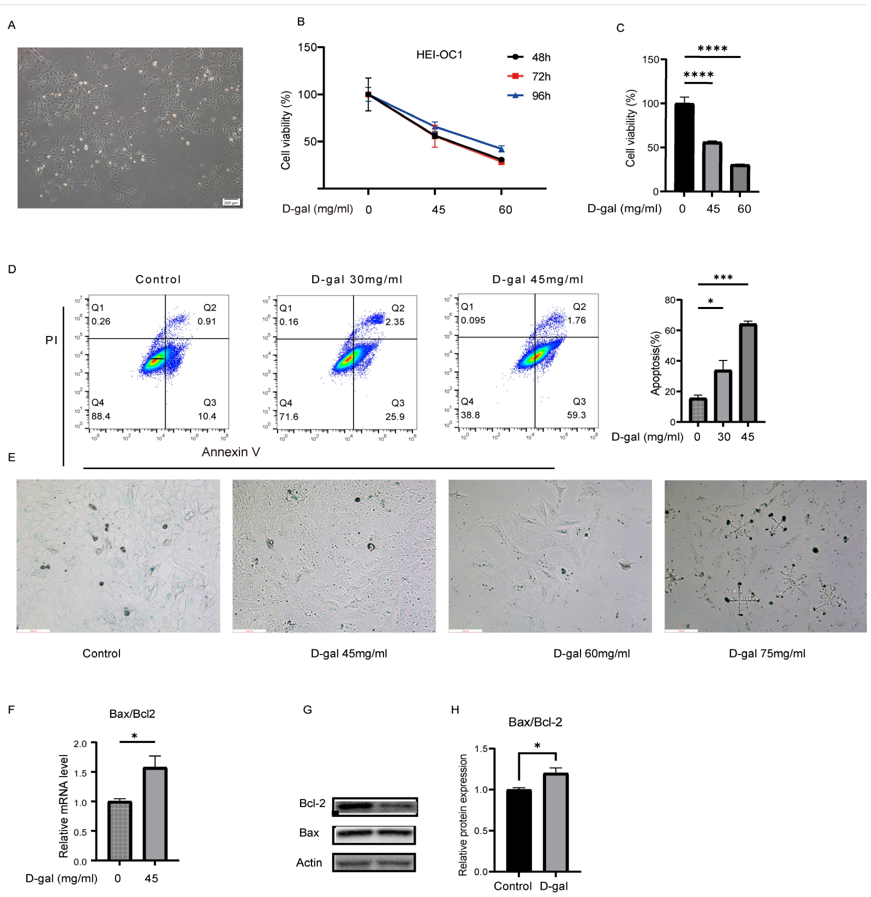

S2. Establishment HEI-OC1 Cells senescent model by D-Galactose

(A) HEI-OC1 cells (4X, scale bar: 200 μm). (B) CCK8 cell viability assay after treating HEI-OC1 cells with different concentrations of D-galactose for 48, 72, and 96 hours. (C) Statistical analysis of cell viability after 48 hours of D-galactose treatment in HEI-OC1 cells (n=6, ****$p < 0.0001$, one way ANOVA with Tukey's multiple comparisons test). (D) Annexin V/PI showed the

HEI-OC1 cells apoptosis after 48 hours of D-galactose treatment (0, 30, 45 mg/ml) (n=3, *p<0.05, ***p<0.001,one way ANOVA with Tukey's multiple comparisons test). (E) β-galactosidase staining of HEI-OC1 cells treated (20X, scale bar: 100 μm). (F) Relative expression of apoptosis-related genes Bax/Bcl-2 increased after D-galactose treatment (n=3,*p<0.05, t test). (G) (H)Western blot showed expression of apoptosis-related proteins Bax and Bcl-2 after D-galactose treatment (n=3,*p<0.05, t test).

S3. Immunofluorescence analysis of CD74 expression in cochlea

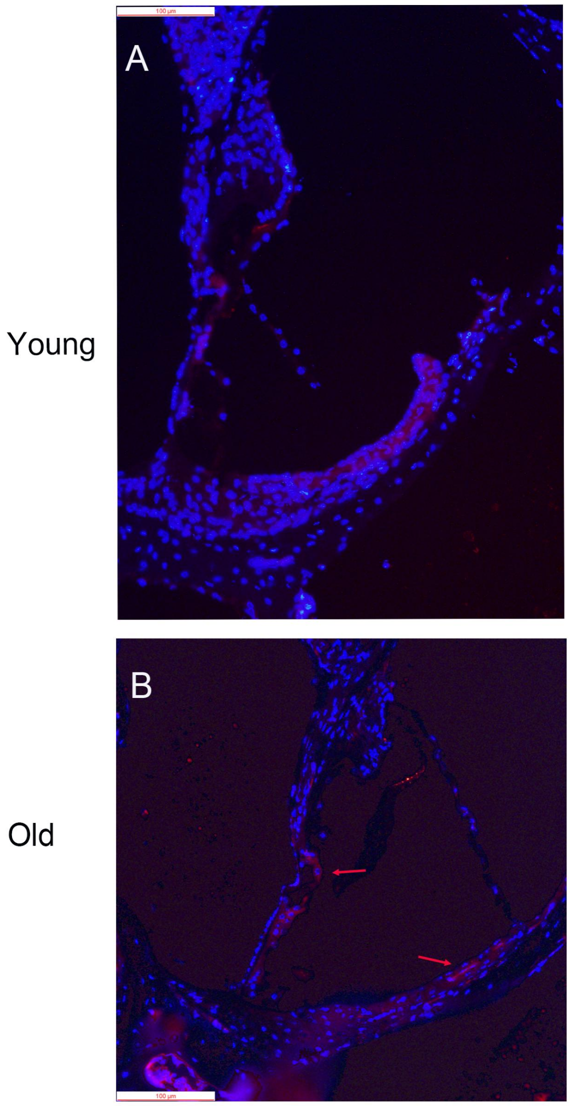

S3. Immunofluorescence analysis of CD74 expression in cochlea

Representative images of CD74 staining (red) in young(A) and aged cochlea (B). Nuclei are counterstained with DAPI (blue).(20X, Scale bar: 100μm.）
